# Genetic variation affects binge feeding behavior in female inbred mouse strains

**DOI:** 10.1038/s41598-019-51874-7

**Published:** 2019-10-31

**Authors:** Brandon A. Newmyer, Ciarra M. Whindleton, Nandan Srinivasa, Marieke K. Jones, Michael M. Scott

**Affiliations:** 1 Department of Pharmacology, University of Virginia, Charlottesville, VA USA; 2 Health Sciences Library, University of Virginia, Charlottesville, VA USA

**Keywords:** Obesity, Genetics of the nervous system

## Abstract

Identifying genetic variants that regulate binge eating (BE) is critical for understanding the factors that control this behavior and for the development of pharmacological treatment strategies. Although several studies have revealed specific genes capable of affecting BE behavior, less is known about how genetic variation modulates BE. Thus, through a paradigm that promoted binge-like food intake through intermittent access to high calorie diet (HCD), we quantified food-intake in four inbred mouse strains: C57Bl/6J (B6), NOD/LtJ (NOD), 129S1/SvlmJ (S1), and A/J (AJ). We report that genetic variation likely influences the chronic regulation of food intake and the binge-like consumption of a palatable HCD. AJ mice consumed more of both standard chow and HCD than the other three strains tested when both diets were available *ad libitum*, while S1 mice consumed significantly less HCD than other strains during intermittent HCD access. Behavioral differences were also associated with differential changes in c-Fos immunohistochemistry in brain regions traditionally associated with appetite regulation. Our results identify 129S1/SvlmJ as a strain that exhibits low levels of binge feeding behavior and suggests that this strain could be useful in the investigation of the influence of genetic variation in the control of binge food intake.

## Introduction

Binge eating (BE) is characterized as compulsive and unrestrained consumption of a large amount of food, typically highly palatable and calorically dense, within a brief time period^1^. While Binge Eating Disorder is recognized as a psychiatric condition in its own right, BE is also often observed in diseases of disordered feeding such as bulimia^2,3^ and in subsets of patients with obesity^4,5^ and anorexia^3^. Identifying genetic variants that regulate BE is critical to both understanding the mechanisms driving this behavior and for the development of pharmacological strategies to combat this condition.

Several gene deletion studies in mice have elucidated specific genes capable of affecting BE behavior: heterozygous cytoplasmic FMR1-interacting protein 2 knockout reduces binge eating of palatable food but not chow consumption in C57BL/6NJ mice^6^, while growth-hormone secretagogue receptor 1A (GHSR) knockout decreased intermittent high fat diet intake in CD-1 mice^7^. Although these and other studies utilizing genetic mouse models have revealed how select factors influence BE, considerably less is known pertaining to what extent natural genetic variation can affect BE behavior.

While differences in binge food intake between C57bl/6J and N lines^6^, in addition to DBA mice^8^, have been described, exhibiting varying levels of binge food intake depending upon the paradigm used to investigate this behavior^9^, we sought to investigate binge-like food intake across several mouse strains using a common experimental approach.

Thus, using a paradigm that involved allowing mice intermittent access to a palatable, high-calorie diet (HCD)^9^, female mice from four inbred lines that form part of the Collaborative Cross population^10^ (C57Bl/6J (B6), NOD/LtJ (NOD), 129S1/SvlmJ (S1), and A/J (AJ)) were tested for BE behavior. When given *ad libitum* access to either chow or HCD in two separate experiments, little variation was observed between strains, with AJ mice consuming significantly more of both diets relative to the other strains tested. Interestingly, during intermittent access to HCD, the four strains showed significantly more variation in binge food intake. NOD, S1, and AJ mice all consumed less HCD than the B6 mice during a 3 h HCD binge, while S1 mice ate significantly less HCD than NOD, and AJ mice. In addition, significant variation in neuronal activation was observed between strains in select brain nuclei shown to modulate feeding behavior, suggesting that gene expression differences within circuits involving these neuronal populations may control binge food intake.

## Methods

Several of the procedures described below have also been reported in our prior publication^11^.

### Animals

Female mice that comprise four of the eight founder strains of the Collaborative Cross^10^ population were obtained from The Jackson Laboratory. We chose to perform this investigation in female animals as the incidence of diseases of disordered feeding is high in females^12^ while significantly less is know about the binge-like food intake performance in female compared to male mice. 8 week old C57Bl/6J (B6), NOD/LtJ (NOD), 129S1/SvlmJ (S1), and A/J (AJ) female mice were acclimated (single-housed) for 1 week upon arrival. 6–7 animals were used from each line in the food intake experiments and in the cFos analysis. Animal use was in accordance with guidelines approved by the University of Virginia Animal Care and Use Committee.

### Measurement of food intake and binge feeding behavior

**Figure 1:**
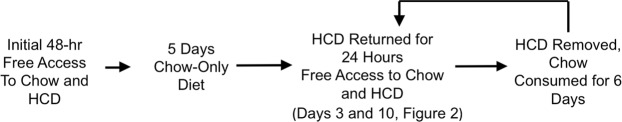
Diagram describing the intermittent feeding paradigm that was used to produce binge-like food intake (modified from Czyzyk *et al*.^9^). Animals in the Intermittent access group first had 48 hr. access to both HCD and chow, after which time, HCD was removed and animals were maintained on chow. Following this period, HCD was reintroduced for 24 hr. Intake was then measured at 3 hr and 24 hr following HCD reintroduction (Fig. 2I–L, Intermittent exposure as presented on the x-axis at “day 3”). Following the initial intermittent exposure period, HCD was then removed and animals were fed chow for 6 days. Following this 6 day period, HCD was returned for 24hrs for a second time (Fig. 2I–L, intermittent exposure as presented on the x-axis at “day 10”). Data from this second intermittent HCD exposure episode are used for all reported analyses.

**Figure 2:**
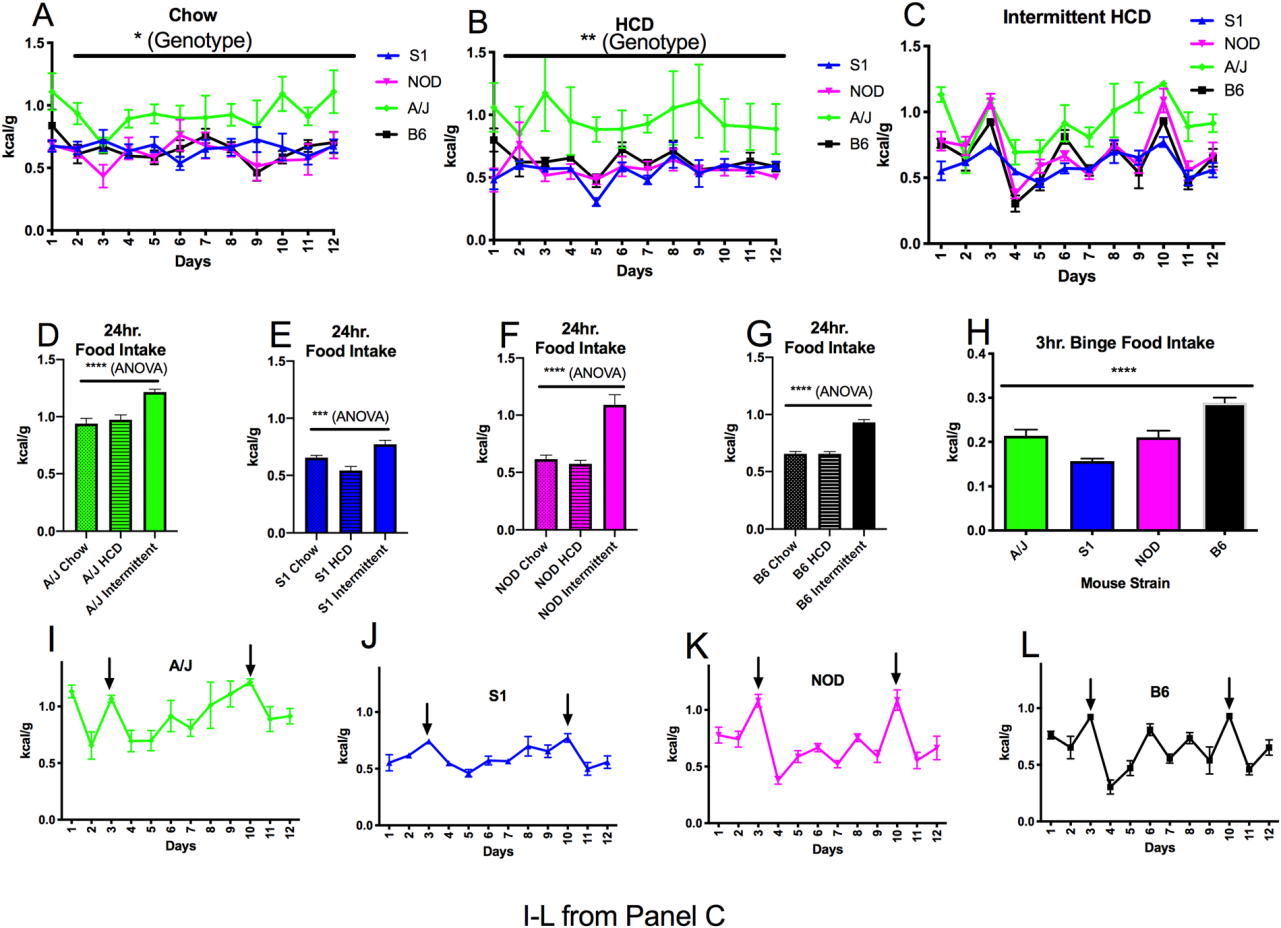
Daily food intake for mouse inbred strains consuming chow (**A**), HCD (**B**) and receiving intermittent access to HCD. (**C**) Significant differences in chow (A, 2-way ANOVA, effect of genotype, F_3,21_ = 6.762, p = 0.0023, effect of time, F_6.5,135.6_ = 2.176, p = 0.0446, interation of genotype x time, F_36,252_ = 1.342, p = 0.1021, Table 1 displays the multiple comparisons between genotypes)) and HCD (B, 2-way ANOVA, effect of genotype, *F*_3,19_ = 4.55, p = 0.0145, effect of time, *F*_3.198,60.77_ = 1.545, *p* = 0.2097, interaction of genotype x time, *F*_33,209_ = 0.8011, *p* = 0.7727, Table 2 displays the multiple comparisons between genotypes) intake were observed between strains. Intermittent exposure in each strain produced elevated intake, relative to continual chow or HCD intake (One way ANOVA with p values reported for the ANOVA and for Dunnett’s post hoc test comparing intermittent intake to chow and to HCD intake, (**D**) ANOVA p < 0.0001, F_2,17_ = 19.19, Dunnett’s p < 0.0001 (chow) p = 0.0004 (HCD), (**E**) ANOVA p = 0.0002, F_2,19_ = 13.52, Dunnett’s p = 0.032 (chow), p = 0.001 (HCD), (**F**) ANOVA p < 0.0001, F_2,15_ = 24.21, Dunnett’s p < 0.0001 (chow) p < 0.0001 (HCD), (**G**) ANOVA p < 0.0001, F_2,17_ = 51.02, Dunnett’s p < 0.0001 (chow), p < 0.0001, (HCD)) Significant variation between strains was also observed following binge feeding during intermittent exposure to HCD (2 H ANOVA, *F*_3,25_ = 25.05, p < 0.0001, multiple comparisons reported in Table 3). Panels I-L are derived from Panel C, demonstrating the timing of the intermittent HCD access/binge feeding episodes (arrows), with data reported from the day 10 intermittent exposure period. 6–7 animals from each genotype were used for each treatment.

Our food intake paradigm was performed as described by Czyzyk *et al*.^9^, avoiding the use of animal stress or food restriction to produce the binge like intake of food (as diagramed in Fig. 1). Briefly, intake of regular chow (Teklad #2014, 4% fat, 17% protein and 48% carbohydrate (no sucrose), 2.9 kcal/g) or HCD (Teklad TD.88137, 21% fat, 48% sucrose, 17% protein 4.5 kcal/g) in the continual, *ad libitum* access groups was weighed daily following a 1-week acclimation period, then averaged over a 7 day period to obtain daily food intake values. In the intermittent food exposure group, mice initially received 48-hour continuous access to both chow and HCD. This initial access period was important to reduce neophobia and novelty for the HCD; parameters that would affect food intake. After 48 hr, the HCD was removed for 5 days while the chow diet was still available *ad libitum*. HCD then was reintroduced in addition to chow, starting 2 hours following the onset of the dark cycle. Chow and HCD were separated by a divider that was part of the metal rack top of the animal’s home cage. Position of the HCD and chow in the metal rack was varied to avoid position effects. Intake was monitored for 3 hours and then 24 hours, following initial HCD presentation (Indicated in Fig. 2I–L by an arrow at “day 3” on the x-axis). We describe this intake as “binge-like” as the animals exhibit the intake of an elevated amount of food relative to their average daily intake, within a brief time period^1^. Following the initial intermittent exposure period, HCD is then removed and animals are fed chow for 6 days. Following this 6 day period, HCD is returned for 24hrs for a second time. Data are presented from this second intermittent HCD exposure episode (Fig. 2. Binge feeding, intermittent exposure to HCD periods demarked by an arrow in Fig. 2I–L, at “day 10” on the x-axis). Food intake was normalized to body weight, as described by Czyzyk *et al*.^9^. Estrous cycle was not determined during testing.

### c-Fos immunohistochemistry

Animals that were naïve for intermittent or constant HCD exposure were presented with 0.1 g of HCD and sacrificed 90 minutes following the onset of consumption. Importantly, all mice showed complete consumption of the small sample of HCD within 30 minutes of presentation. Mice were anesthetized i.p. with euthanasia solution (0.1 ml euthasol) and underwent trans-cardiac perfusion with buffered saline followed by fixative solution (4% paraformaldehyde in 0.1 M phosphate buffer, pH 7.4, containing 15% saturated picric acid). Each mouse was perfused with 50 ml fixative over a 5 min time period. Following perfusion, brains were dissected and post-fixed in the same fixative solution overnight, after which they were transferred to 0.1 M phosphate buffer. Brains were blocked coronally into three equally sized sections using a mouse brain mold. The brain parts were blotted, dried and arranged into standard cryomolds (Tissue Tek, #4557) with brain sections from four to six different mice in each cryomold. The molds were then filled with warmed 10% gelatin solution, allowed to cool to solidify, and post-fixed in 4% paraformaldehyde solution at 4 °C overnight. The blocks were then removed from molds, trimmed, glued on polystyrene dishes, and cut into coronal sections (40 μm thick) using a vibratome (Leica). The sections were collected serially in six-well tissue culture plates such that each well contained a representative series with every 6th section present (distance between adjacent sections in each well was therefore 240 μm). Sections were stored at 4 °C in 0.1 M phosphate buffer containing 0.1% sodium azide as a preservative prior to immunohistochemical procedures.

Sections were stained for c-Fos immunoreactivity using peroxidase staining^11,13^. Phosphate-buffered saline (PBS) was used for all rinses, whereas all antibody solutions were made in PBS containing 0.5% Triton X-100, 0.1% sodium azide, and 2% normal goat serum. First, sections were pretreated with sodium borohydride (0.1%) in PBS for 20 min followed by immersion into 0.3% hydrogen peroxide and 0.1% sodium azide in PBS (30 min) to quench endogenous peroxidase activity. Next, the sections were immersed in blocking solution containing 2% normal goat serum and Fab’ fragments of goat anti-mouse IgG (1:1000) for 4 h at room temperature. Sections were then incubated in anti-c-Fos (Ab5, EMD Millipore, #PC38, 1:50,000) for 72 h followed by overnight incubation in biotinylated goat anti-rabbit IgG (Jackson ImmunoResearch, 1:1000) with antibodies diluted in PBS containing 0.5% Triton X-100 and 0.1% sodium azide. Subsequently the sections were immersed in avidin– biotin–peroxidase complex diluted in PBS with 0.1% Triton X-100 (ABC Elite kit, Vector; 1:1000, 4 h). Staining was completed using nickel-enhanced 3,3’ -diaminobenzidine (DAB, 0.02%, nickelous ammonium sulfate 0.15%) in Tris–HCl (0.05 M, pH 7.6) yielding a black reaction product.

### Microscopy

The sections were examined with an Olympus BX51 microscope using 10, 20, and 40× objectives and digital images were captured using a Magnafire digital camera (Optronics, Goleta, CA, USA) and stored images in TIFF format or loaded into NIH Image (version 1.61) for counting cell nuclei stained for c-Fos immunoreactivity.

### Counting of Fos+ cells

Quantitation of c-Fos expression was done using NIH Image (v.1.61). Images were first processed by equalizing brightness (background subtraction), and were then thresholded for binary conversion. The number of particles (which corresponded to labeled nuclei) were then counted and recorded. For each brain area investigated, we chose two sections that showed well defined nuclei prominently present, and the number of c-Fos-labeled cells were counted bilaterally in each section and summated to yield the total number. We performed cell counts from nuclei centered on the following stereotaxic coordinates relative to mouse Bregma, as determined from the atlas of Paxinos and Franklin^14^. Medial (MO), ventral (VO) orbitofrontal cortex +3.08 mm, prelimbic (PL) and infralimbic (IL) cortex (combined to form the PFC cell counts) +1.8 mm, granular gustatory (Gran) cortex +1.8 mm, rostral accumbens (Acb) +1.54 mm, lateral hypothalamus (LH) −1.58 mm, arcuate nucleus (Arc) −1.58 mm, Ventral Tegmental Area (VTA) −3.16 mm, central nucleus of the amygdala (CeA) −1.22 mm, medial parabrachial nucleus (PB) −5.20 mm, nucleus of the solitary tract (NTS) −7.2 mm.

### Statistical analysis

The amount of food intake consumed was analyzed using either a two-way or a one-way ANOVA with Tukey’s or Dunnett’s multiple comparisons test in GraphPad Prism (ver.6). All treatment groups were composed of 6–7 animals per group. Correlations between the intake of chow, HCD intake during continual exposure and HCD intake during intermittent exposure were performed in Graphpad Prism (ver.6). Analysis of c-Fos expression differences between genotypes were analyzed using multiple ANOVAs followed by Benjamini-Hochberg p-value correction for multiple comparisons, performed in R (ver. 3.5.3). All grouped values in the bar graphs are expressed as means and standard error of the mean (SEM). Differences with p < 0.05 were considered statistically significant. Graphs were prepared in Graphpad Prism (ver.6).

## Results

We investigated whether four inbred strains of female mice showed differences with respect to the ingestion of chow or HCD under constant or intermittent access conditions, with the intermittent exposure paradigm diagramed in Fig. 1. Food intake for each individual strain during this paradigm is presented in Fig. 1I–L, with each intermittent HCD exposure period demarked by arrows, on days 3 and 10. Data from the second intermittent exposure period, at day 10, were used for analysis. While the S1, NOD and B6 strains all showed similar levels of chow and HCD consumption, A/J female mice showed an elevation in intake relative to the other mouse strains (Fig. 2A, 2-way ANOVA, effect of genotype, F_3,21_ = 6.762, p = 0.0023, effect of time, F_6.5,135.6_ = 2.176, p = 0.0446 interaction of genotype x time, F_36,252_ = 1.342, p = 0.1021, Table 1 reports the multiple comparisons between genotypes) and also exhibited an increase in HCD intake (Fig. 2B, 2-way ANOVA, effect of genotype, *F*_3,19_ = 4.55, p = 0.0145, effect of time, *F*_3.198,60.77_ = 1.545, *p* = 0.2097, interaction of genotype x time, *F*_33,209_ = 0.8011, *p* = 0.7727, Table 2 reports the multiple comparisons between genotypes). In addition to the elevated intake seen in A/J females, S1 mice showed reduced HCD intake when compared to B6 animals (Table 2). Mice undergoing the intermittent feeding paradigm all showed elevated intake of food during the intermittent exposure to HCD, when compared to daily intake seen in control groups of the same genotype that were either continually fed chow or HCD. (One way ANOVA with p values reported for the ANOVA and for Dunnett’s post hoc test comparing intermittent intake to chow and to HCD intake, Fig. 2D, ANOVA p < 0.0001, F_2,17_ = 19.19, Dunnett’s p < 0.0001 (chow) p = 0.0004 (HCD), Fig. 2E, ANOVA p = 0.0002, F_2,19_ = 13.52, Dunnett’s p = 0.032 (chow), p = 0.001 (HCD), Fig. 2F, ANOVA p < 0.0001, F_2,15_ = 24.21, Dunnett’s p < 0.0001 (chow) p < 0.0001 (HCD), Fig. 2G, ANOVA p < 0.0001, F_2,17_ = 51.02, Dunnett’s p < 0.0001 (chow), p < 0.0001, (HCD)).

**Table 1:** Statistical table describing the 2-way ANOVA output and the multiple comparisons made in Fig. 1, panel A, between mouse strains during chow food intake.

ANOVA table	SS	DF	MS	F(DFn,DFd)	P value
Time x Genotype	1.260	33	0.03819	F(33,209) = 0.8011	P = 0.7727
Time	0.8103	11	0.07367	F(3.198,60.77) = 1.545	P = 0.2097
Genotype	8.154	3	2.718	F(3,19) = 4.55	P = 0.0145
Residual	9.964	209	0.04767		
**Tukey’s multiple comparison’s test**	**Mean Diff.**	**95% CI of Diff.**	**Adjusted P value**		
S1 vs NOD	−0.01582	−0.09141 to 0.05977	0.9476		
S1 vs A/J	−0.4188	−0.5819 to −0.2558	<0.0001		
S1 vs B6	−0.08378	−0.1526 to −0.01497	0.0101		
NOD vs. A/J	−0.4030	−0.5693 to −0.2368	<0.0001		
NOD vs B6	−0.06796	−0.1447 to 0.008760	0.1020		
A/J vs B6	0.3351	0.1716 to 0.4986	<0.0001

**Table 2:** Statistical table describing the 2-way ANOVA output and the multiple comparisons made in Fig. 1, panel B, between mouse strains during HCD intake.

ANOVA table	SS	DF	MS	F(DFn,DFd)	P value
Time x Genotype	1.639	36	0.04552	F(36,252) = 1.34	P = 0.1021
Time	0.8861	12	0.07384	F(6.456,135.6) = 2.16	P = 0.0446
Genotype	4.834	3	1.611	F(3,21) = 6.762	P = 0.0023
Residual	8.550	252	0.03393		
**Tukey’s multiple comparison’s test**	**Mean Diff.**	**95% CI of Diff.**	**Adjusted P value**		
S1 vs NOD	0.03131	−0.04737 to 0.1100	0.7300		
S1 vs A/J	−0.2750	−0.3780 to −0.1720	<0.0001		
S1 vs B6	−0.003454	−0.07503 to 0.06812	0.9993		
NOD vs. A/J	−0.3063	−0.4160 to −0.1967	<0.0001		
NOD vs B6	−0.03476	−0.1159 to 0.04634	0.6818		
A/J vs B6	0.2716	0.1668 to 0.3764	<0.0001		

**Figure 3:**
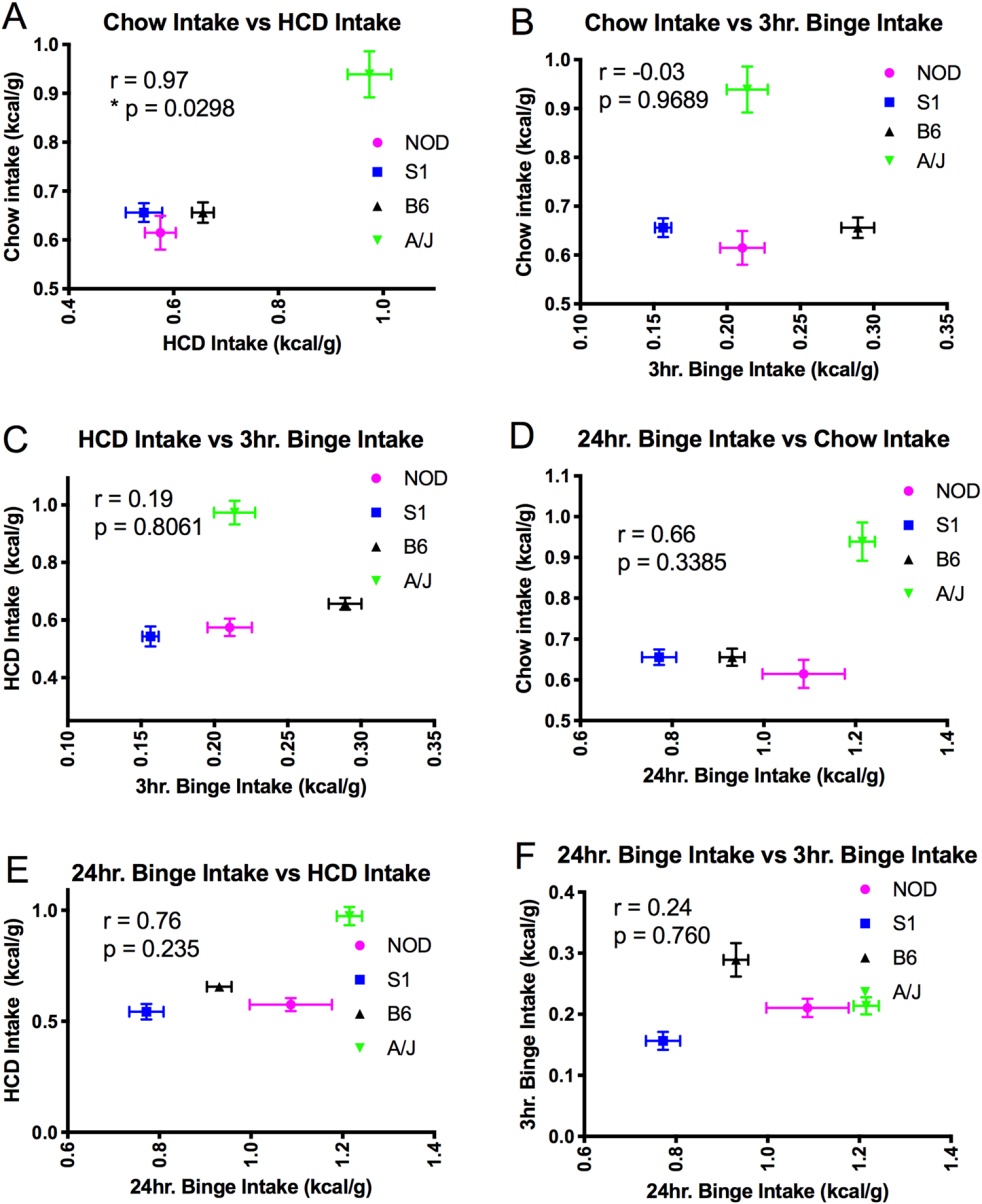
(**A**) Chow food intake and HCD diet intake show high levels of correlation across mouse strains (r = 0.97, p = 0.298) Binge food intake shows little correlation with chow intake (**B**,**D**) or with HCD intake (**C**,**E**). Acute versus chronic feeding in the intermittent exposure to HCD group also shows little correlation across mouse strains (**F**).

Interestingly, when mouse strains had intermittent access to HCD, the resulting binge-like, elevated intake of HCD did not occur to the same extent across all strains (Fig. 2H). While all inbred strains showed an average intake during the 3 hour binge-like feeding episode that was less than that of the B6 strain, the S1 animals consumed less than all other strains, showing a significant reduction in binge-like food intake (Fig. 2H, ANOVA, *F*_3,25_ = 25.05, p < 0.0001 with multiple comparisons reported in Table 3). These data suggest that genetic variation between the strains significantly affects binge feeding behavior. We then wanted to determine whether the regulation of *ad libitum* food intake in these inbred strains correlated with intermittent food intake, as prior studies suggest that the neuronal mechanisms that regulate food intake differ between these two behavioral paradigms^9^. As expected, intake in the *ad libitum* fed chow and HCD groups was highly correlated (Fig. 3A, r = 0.97, p = 0.0298, suggesting that similar neuronal systems may be involved in determining intake based on the caloric content of food. However, neither HCD nor chow intake correlated with the food intake of intermittent HCD exposed animals, either at 3 hr (Fig. 3B r = −0.03, p = 0.969, Fig. 3C r = 0.19, p = 0.806) or 24 hr (Fig. 3D r = 0.3385, p = 0.338, Fig. 3E r = 0.235, p = 0.235) time points. Thus, our work suggests that binge food intake and the regulation of food intake based on caloric value may be regulated by separate mechanisms that are affected by the genetic variation observed between the S1, NOD, B6 and AJ inbred mouse strains.

**Table 3:** Statistical table describing the multiple comparisons made (1-way ANOVA, Tukey’s post hoc test) in Fig. 1, panel H, between mouse strains during the 3 hr intermittent exposure to HCD.

Tukey’s Multiple Comparisons Test	Mean Diff.	95% CI or Diff.	Adjusted P value
S1 vs NOD	−0.05397	−0.09971 to −0.008228	P < 0.05
S1 vs AJ	−0.05724	−0.1011 to −0.01341	P < 0.01
S1 vs B6	−0.1327	−0.1750 to −0.09033	P < 0.0001
NOD vs AJ	−0.003271	−0.05039 to 0.04385	0.9975
NOD vs B6	−0.07871	−0.1245 to −0.03297	P < 0.001
AJ vs B6	−0.07544	−0.1193 to −0.03160	P < 0.001

**Figure 4:**
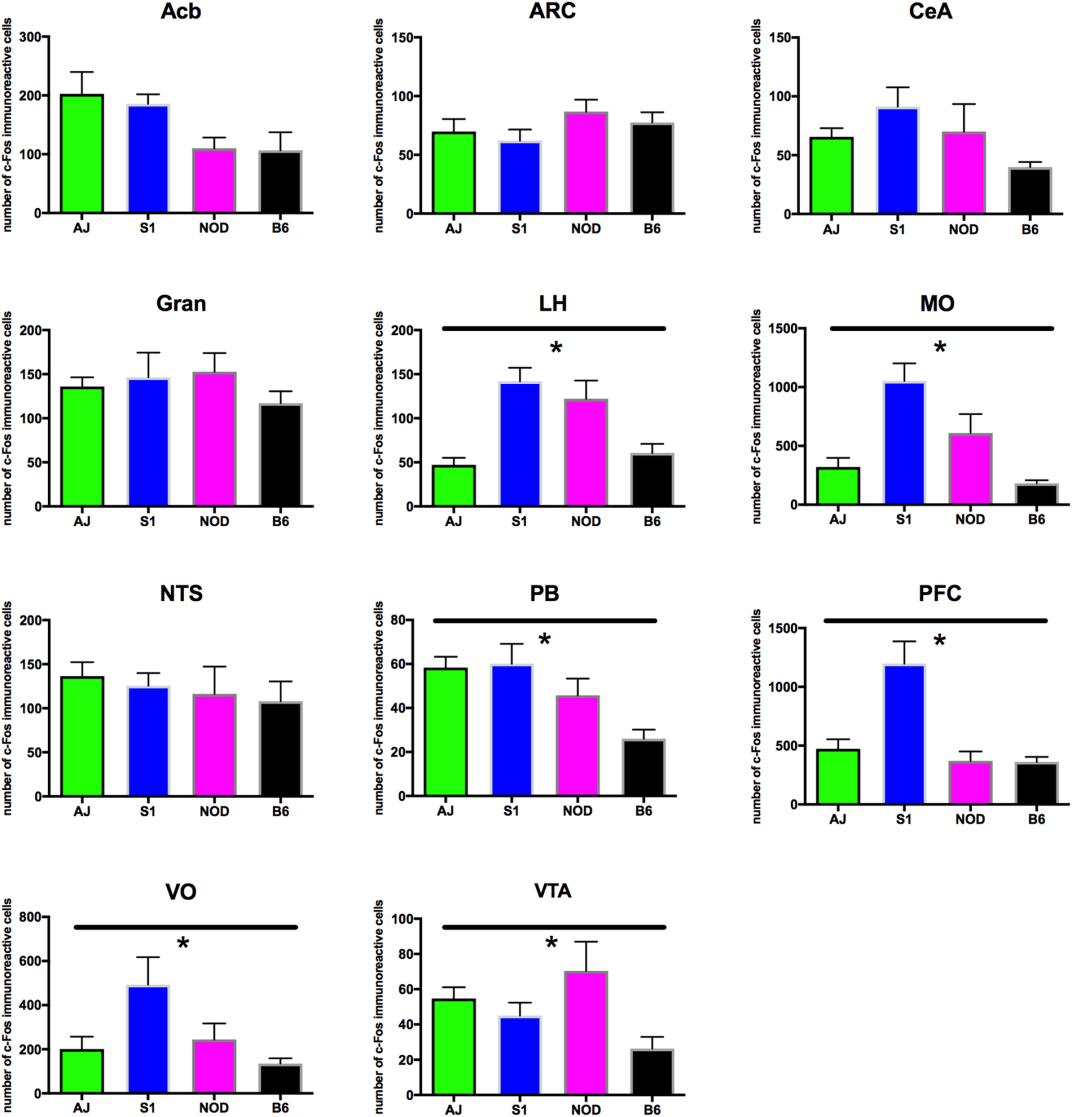
cFos expression in response to HCD feeding. Animals were fed 0.1 g HCD 90 minutes prior to sacrifice, with all animals consuming the test diet. cFos expression was then determined in brain areas shown previously to regulate food intake behavior. While cFos expression does not show variation between mouse strains in select brain nuclei (A, Acb, B, Arc, C, CeA, D, Gran and G, NTS). cFos expression in the LH (E), MO (F), PB (H), PFC (I), VO (J), VTA (K) all exhibited genotype dependent differences (ANOVA, p < 0.05, Benjamini-Hochberg p-value correction for multiple comparisons).

**Figure 5:**
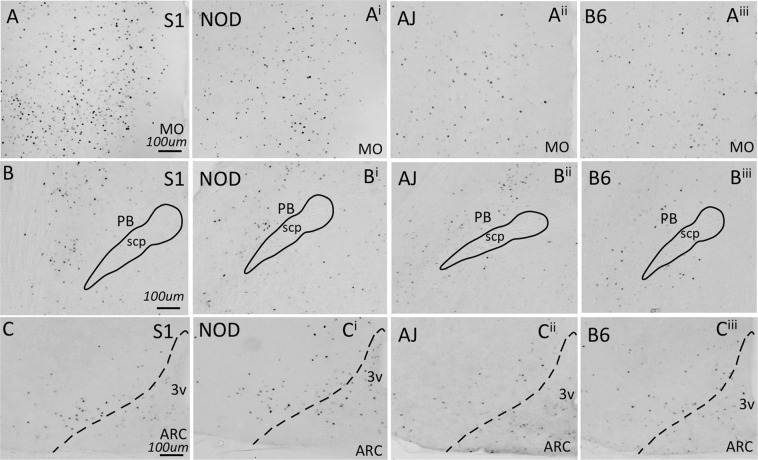
Examples of cFos expressing brain areas from all four mouse strains challenged with HCD, as quantified in Fig. 3. Increased cFos expression was observed in the MO (**A**) and PB (**B**) across mouse lines, while little change in cFos expression was observed in the Arc (**C**).

To begin to characterize whether neuronal activation within brain regions shown to regulate feeding is dependent upon strain genotype, we examined c-Fos expression following the ingestion of a small quantity (0.1 g) of HCD in naïve animals. Several brain areas exhibited genotype-dependent expression of c-Fos. The lateral hypothalamus (ANOVA, *F*_3,24_ = 13.55, p = 1.23 × 10^−4^), medial- (ANOVA, *F*_3,23_ = 14.46, p = 1.23 × 10^−4^) and ventral orbitofrontal cortices (ANOVA, *F*_3,23_ = 4.028, p = 0.0379), medial prefrontal cortex (ANOVA, *F*_3,25_ = 11.75, p = 1.98 × 10^−4^), ventral tegmental area (ANOVA, *F*_3,22_ = 3.992, p = 0.0379), and parabrachial nucleus (ANOVA, *F*_3,23_ = 6.856, p = 0.00502) all showed significant differences in c-Fos expression across genotype (Fig. 4). However, several other brain areas that have been demonstrated to regulate food intake exhibited no differences in c-Fos expression across the genotypes tested (Fig. 4): the arcuate nucleus (ANOVA, *F*_3,25_ = 1.017, p = 0.468), nucleus accumbens (ANOVA, *F*_3,24_ = 2.978, p = 0.07189) central nucleus of the amygdala (ANOVA, *F*_3,25_ = 2.947, p = 0.07189), granular cortex (ANOVA, *F*_3,18_ = 0.9769, p = 0.468) and caudal nucleus of the solitary tract (ANOVA, *F*_3,25_ = 0.3978, p = 0.756). Differences in c-Fos expression can be readily observed in images presented from the S1, NOD, AJ and B6 strains, of sections from medial orbitofrontal cortex and parabrachial nucleus (Fig. 5A,B). Little difference in expression can be seen across genotype within the arcuate nucleus (Fig. 5C). Finally, we looked at whether differences in cFos expression in select brain nuclei correlated with variation in 3 hr. binge-like food intake observed between the four inbred mouse strains. As we describe in Table 4, however, no significant correlations were observed.

**Table 4:** Correlations between cFos expression in select brain regions and 3 hr. binge-like food intake levels in the inbred mouse strains.

Brain Region	Acb	Arc	CeA	Gran	LH	MO	NTS	PB	PFC	VO	VTA
Pearson r	−0.639	0.521	0.873	−0.82	−0.704	−0.9	−0.615	−0.909	−0.784	−0.893	−0.515
95% Confidence interval	−0.9913 to 0.8347	−0.8814 to 0.9876	−0.5478 to 0.9973	−0.9961 to 0.6666	−0.9931 to 0.7950	−0.9979 to 0.4538	−0.9906 to 0.8462	−0.9981 to 0.4137	−0.9952 to 0.7188	−0.9978 to 0.4794	−0.9874 to 0.8833
P value	0.361	0.479	0.127	0.181	0.296	0.100	0.385	0.0913	0.217	0.107	0.485

## Discussion

Our data suggest that genetic variation likely influences the regulation of excessive food intake over short periods of time. Minimal variation in food intake (either of chow or palatable diet) during constant, *ad libitum* access was observed between strains, with the A/J strain showing the largest increased consumption relative to other mice. However, the four strains tested showed markedly larger variation in levels of binge-like intake of HCD. Future studies, meanwhile, will be required to determine how the caloric content of the food is responsible for driving the binge-like consumption. Indeed, while chow- and HCD intake between mouse strains showed a high level of correlation, no correlation was observed between intermittent, binge-like intake and the levels of total food intake during *ad libitum* access conditions. These data then suggest that the genetic control of consumption during continual access to chow or palatable food is possibly controlled by similar genetic factors. Lack of a correlation between binge food intake during intermittent HCD exposure and food intake during continual exposure to either HCD or chow suggests that the genetic factors that control binge intake differ from those that regulate food intake during continual exposure.

Interestingly, the lack of a significant correlation between cFos expression in select brain nuclei and variation in binge-like food intake suggests that the predictive power of single nuclei cFos activity is weak and that the genetic effects driving variation in binge-like food intake are likely llikely spread over multiple brain areas. It should be noted, however, that testing further mouse strains would help strengthen this interpretation, as our work was based on the analysis of just four of the commonly used inbred strains available at The Jackson Laboratory.

The identification of inbred mouse strains that show significant variation in binge food intake under an intermittent exposure paradigm will be extremely useful in the investigation of genetic variants that influence excessive reward-seeking behavior. While mouse strains such as DBA/2j exhibit high levels of binge food intake in certain paradigms^8^, our data using an intermittent exposure model system describes how inbred lines, most notably the 129S1/SvlmJ line, exhibit significantly reduced binge food intake, relative to these other commonly used strains. Consequently, the description of mouse strains that show both excessive and constrained binge feeding could significantly increase our ability to identify the genetic networks that drive aberrant food- and possibly drug intake behavior.

Significant variation between 129 substrains and the C57Bl/6j strain have been reported in prior work investigating both reward seeking and reward potency. For example, food and cocaine reinforcement in addition to place preference for cocaine was reduced in 129X1/SvJ animals compared to C57bl/6J mice^15,16^. Morphine, however, was significantly more potent in producing analgesia in 129 substrains compared to C57Bl/6 mice^17^. DBA animals, meanwhile, were shown to lack any sensitivity towards the rewarding effects of morphine unlike that observed with C57Bl/6j mice^18^. This variation in response to morphine is especially relevant, given that prior work has demonstrated the importance of the μ-opioid receptor in driving binge feeding behavior^19,20^. Our study therefore demonstrates that, in addition to exhibiting significantly different responses to drugs of abuse, these strains also exhibit differences in binge-like consumption of natural rewards.

The diversity outbred (DO)^21^ and collaborative cross (CC)^10^ populations both include the founder strains investigated here, suggesting that these mouse populations should be extremely useful for the study of binge feeding genetics. In addition to the inbred strains, the DO and CC also include wild-derived founder animals. Although we attempted to investigate whether one of these wild derived strains, the Cast/EiJ line^10^, exhibits binge food intake, we found it impossible to measure consumption of the TD.88137 diet. Whether as a pellet or in a paste formulation, the Cast/J mice would disperse the food around their cage in small fragments, making measurement of intake imprecise. Future work will focus on investigating how the wild-derived and inbred strains tested in the current report differ with respect to binge food intake, using a modified binge food intake paradigm.

When we examined the brain regions activated following exposure to calorically dense food, significant differences in c-Fos expression were observed in several brain regions involved in food intake regulation based on palatability^22–24^. Significantly less of an effect on c-Fos expression was observed in brain regions shown to regulate food intake based on the metabolic requirement of the animal, similar to data reported previously^25^. Although it is difficult to conclude that our observed c-Fos expression differences reflect changes in the activity of brain areas that drive observed strain-dependent differences in binge food intake, our work is highly suggestive of this possibility, as the rank order of c-Fos expression in many frontal cortical brain areas reflects a similar rank order in levels of HCD intake. Whether the differences in cFos expression arise solely as a result of the exposure of the animal to HCD or reflect activity levels present in the absence of HCD stimulation will require further investigation.

Both the prefrontal and orbitofrontal cortices, brain areas that showed significant differences in c-Fos expression between strains, have previously been shown to play important roles in driving binge feeding behavior. In rodents, μ-opioid receptor activation has been shown to be both necessary and sufficient^19,20^ in these brain areas to affect binge-like food intake. Optogenetic manipulation of projections from the frontal cortex to the amygdala, meanwhile, has revealed this subcortical projection to be both necessary and sufficient to regulate feeding behavior^26^. Additionally, changes in brain activity in medial prefrontal regions is often observed in fMRI studies in humans with altered food intake behavior. For example, reduced blood flow to fronto-striatal circuits is frequently seen in people exhibiting eating disorders, suggesting that alterations in these brain regions could drive changes in both feeding behavior and impulsive action^2,27^. Furthermore, people who show successful weight loss following the development of obesity demonstrate alterations in the activation of brain areas that regulate sensory processing, reward and impulsivity, when compared to overweight controls^28^.

In conclusion, our work demonstrates how genetic variation may directly or indirectly affect the activity of brain regions shown to regulate feeding behavior. We also suggest that the mouse strains tested in the current study could be utilized to map genetic variants that could contribute to the development of disordered feeding.

## Data Availability

The data sets generated during the current study are available from the corresponding author on reasonable request.
