# Publisher Correction: Genetic variation affects binge feeding behavior in female inbred mouse strains

**DOI:** 10.1038/s41598-020-57579-6

**Published:** 2020-01-14

**Authors:** Brandon A. Newmyer, Ciarra M. Whindleton, Nandan Srinivasa, Marieke K. Jones, Michael M. Scott

**Affiliations:** 1 Department of Pharmacology, University of Virginia, Charlottesville, VA USA; 2 Health Sciences Library, University of Virginia, Charlottesville, VA USA

Correction to: *Scientific Reports* 10.1038/s41598-019-51874-7, published online 31 October 2019

The Acknowledgements section in this Article was omitted. The Acknowledgements section should read:

“This project was supported by funds received from the NIH, awarded to MMS (1R01MH116694-01), and to BAN (T32 GM007055)”

